# Postmenopausal Women Have Higher HDL and Decreased Incidence of Low HDL than Premenopausal Women with Metabolic Syndrome

**DOI:** 10.3390/healthcare4010020

**Published:** 2016-03-16

**Authors:** Maria Luz Fernandez, Ana Gabriela Murillo

**Affiliations:** Department of Nutritional Sciences, University of Connecticut, Storrs, CT 06269, USA; ana.murillo_solis@uconn.edu

**Keywords:** postmenopausal women, premenopausal women, HDL cholesterol, age, waist circumference, body mass index

## Abstract

It is well known that plasma lipids, waist circumference (WC) and blood pressure (BP) increase following menopause. In addition, there is a perceived notion that plasma high-density lipoprotein-cholesterol (HDL-C) concentrations also decrease in postmenopausal women. In this cross-sectional study, we evaluated plasma lipids, fasting glucose, anthropometrics and BP in 88 post and 100 pre-menopausal women diagnosed with metabolic syndrome. No differences were observed in plasma low-density lipoprotein-cholesterol cholesterol, triglycerides, fasting glucose or systolic and diastolic BP between groups. However, plasma HDL-C was higher (*p* < 0.01) in postmenopausal women and the percentage of women who had low HDL (<50 mg/dL) was higher (*p* < 0.01) among premenopausal women. In addition, negative correlations were found between WC and HDL-C (*r* = −0.148, *p* < 0.05) and BMI and HDL-C (*r* = −0.258, *p* < 0.01) for all subjects indicating that increases in weight and abdominal fat have a deleterious effect on plasma HDL-C. Interestingly, there was a positive correlation between age and plasma HDL-C (*r* = 0.237 *p* < 0.01). The results from this study suggest that although HDL is decreased by visceral fat and overall weight, low HDL is not a main characteristic of metabolic syndrome in postmenopausal women. Further, HDL appears to increase, not decrease, with age.

## 1. Introduction

Menopause is defined as the cessation of menses due to estrogen deficiency for at least one year [1,2], which in North America happens at a median age of 51 [3]. Post-menopausal women may experience a variety of symptoms, including hot flashes, sweating, insomnia, and vaginal dryness and general discomfort [1,4]. In addition, most women undergo physiological changes in the first years following their final menstrual period [3].

One of the physiological changes that occurs during menopause is an increase in body weight [3,5,6,7]. Although the normal aging process is associated with a decreased lean body mass and a slower metabolic rate regardless of sex or hormonal status [6], postmenopausal women tend to experience an accelerated weight gain during the first years of endogenous estrogen decline [5,8]. In addition to overall weight gain, menopause is also linked to changes in body composition and fat distribution [9]. With increasing postmenopausal age, lean body mass decreases while fat mass increases [5,6], predominantly in the abdominal area [3,6,10,11]. This augmented visceral adipose tissue (VAT) is positively associated with cardiovascular risk factors and metabolic diseases [12,13] and it is considered one of the components of the metabolic syndrome (MetS) [14].

MetS is a cluster of clinical criteria used to identify patients at increased risk for cardiovascular disease (CVD), type II diabetes mellitus (T2DM) and all-cause mortality. These risk factors are hypertension (≥130/85 mm Hg), decreased high-density lipoprotein- cholesterol (HDL-C) (<40 mg/dL for men and <50 mg/dL for women) hypertriglyceridemia (triglycerides >150 mg/dL), high fasting blood glucose (>100 mg/dL) and abdominal obesity (waist circumference of ≥102 cm for males and ≥88 cm for females) [15,16]. Increased weight and obesity lead to higher prevalence of MetS in postmenopausal women and this may explain, in part, why there is a twofold increase in risk for CVD after menopause [8,14,17].

Another physiological change that occurs during menopause is a shift towards a more atherogenic lipid profile [8,14]. After the cessation of menses, researchers have found an increase in plasma triglycerides (TG), total cholesterol (TC) and low-density lipoprotein-cholesterol (LDL-C), especially the small and dense particles which are more pro-atherogenic [8,14,18,19].These changes seem to be independent of age and more related to the increased abdominal fat mass [8].

An important component of the lipid profile is HDL-C concentration and size of HDL particles [20]. Clinical and epidemiological data have provided evidence of an inverse relationship between low concentration of HDL-C and an increased risk for CVD [21]. HDL particles not only mediate reverse cholesterol transport (RCT) but also exhibit anti-oxidant, anti-inflammatory, anti-thrombotic and vasodilatory activities [22,23,24]. The changes in the concentration of HDL-C and HDL particles composition after menopause have been subject of controversy. While some authors state that there is a decrease of HDL-C in post-menopausal women [8,14,19], others have reported no changes [25] or even an increase in HDL-C after menopause [26,27]. In the present study, the MetS parameters of premenopausal (*n* = 100) and post-menopausal women (*n* = 88) who were recruited from 2009 to 2015 as participants of different interventions are compared at baseline to analyze the differences observed between these two groups. We hypothesized that HDL-C would not be lower in postmenopausal women and that abdominal fat would be a better predictor of plasma HDL-C concentrations.

## 2. Materials and Methods

### 2.1. Subjects

We analyzed the baseline characteristics of pre (*n* = 100) and post-menopausal women (*n* = 88) who were recruited from 2009 to 2015 as participants of interventions targeted at metabolic syndrome [28,29,30,31]. In the current study, we evaluated plasma lipids, anthropometrics, plasma glucose and blood pressure at baseline (prior to the intervention) to compare these parameters between groups. All human studies were approved by the University of Connecticut Institutional Research Board and all subjects signed an informed consent to participate.

### 2.2. Anthropometrics and Blood Pressure

Body weight was recorded to the nearest 100 g on a calibrated digital scale, with subjects wearing only light clothing. Height was measured to the closest cm. Both height and weight were used to calculate BMI as kg/m^2^. Waist circumference (WC) was measured by using a non-flexible tape, which was placed directly on the skin with the participant having feet together and arms hanging freely. All WC measurements were done to the nearest 0.1 cm immediately above the iliac crest and repeated 3 times for a precise average measurement. Blood pressure was measured on the left arm using an Omron HEM-711 DLX automated blood pressure monitor (Omron healthcare, Inc., Bannockburn, IL, USA) with subjects seated, following a 5–10 min rest.

### 2.3. Blood Sample Collection

After a 12-h overnight fast, blood samples were drawn from each subject. A total of 60 mL blood was collected from the antecubital vein into EDTA- containing tubes to prevent coagulation. Plasma was separated by centrifugation at 2000× *g* for 20 min at 4 °C. Subsequently, phenylmethylsulfonyl fluoride (0.1 mL/100 mL), sodium azide (0.1 mL/100 mL) and aprotinin (0.5 mL/100 mL) were added for preservation after plasma was separated from red blood cells. Plasma samples were then aliquoted and frozen at −80°C until further analysis.

### 2.4. Plasma Lipids and Glucose

Plasma total cholesterol, HDL-C, TG and glucose were all measured using an automated clinical chemistry analyzer (Cobas C 111, Roche Diagnostics, Indianapolis, IN, USA) or via enzymatic methods. Plasma LDL-cholesterol (LDL-C) was estimated by the Friedewald equation [32]. None of the participants had plasma TG > 400 mg/dL thus the calculation of LDL-C by the Friedwald equation was appropriate.

### 2.5. Statistical Analysis

All values are presented as mean ± SD. Un-paired *t*-tests were used to compare pre and postmenopausal women in the measured parameters. When women were classified in low and HDL cholesterol groups, a two-way ANOVA was used to evaluate menopause effect, low HDL effect and interaction. Pearson correlations were done to correlate between anthropometrics and HDL cholesterol and age and plasma HDL-C. A *p* value < 0.05 was considered to be significant.

## 3. Results

### Age, Plasma Lipids, Plasma Glucose, Anthropometrics and Blood Pressure

The values for age, plasma lipids, glucose and anthropometrics are indicated in Table 1. There were no significant differences in body weight, BMI, WC, blood pressure, total cholesterol, LDL cholesterol, triglycerides or glucose between pre and postmenopausal women. Age was different (*p* < 0.001) as expected due to menopausal status. Surprisingly, HDL cholesterol was higher (*p* < 0.001) in postmenopausal women by 11%. Further, the number of women who had low HDL cholesterol was higher in pre *vs.* postmenopausal women (Table 1). The range of ages were 26–49 years for pre-menopausal and 51–74 years for postmenopausal women.

Although there were no significant differences in MetS parameters between pre- and post-menopausal women, the most consistent parameters contributing to this classification were WC, plasma TG and HDL-C for premenopausal women and WC, plasma TG and blood glucose for postmenopausal women. Interestingly the majority of the subjects only had three parameters of MetS and only two in each group (pre and post-menopausal) had 5 MetS parameters.

**Figure 1:**
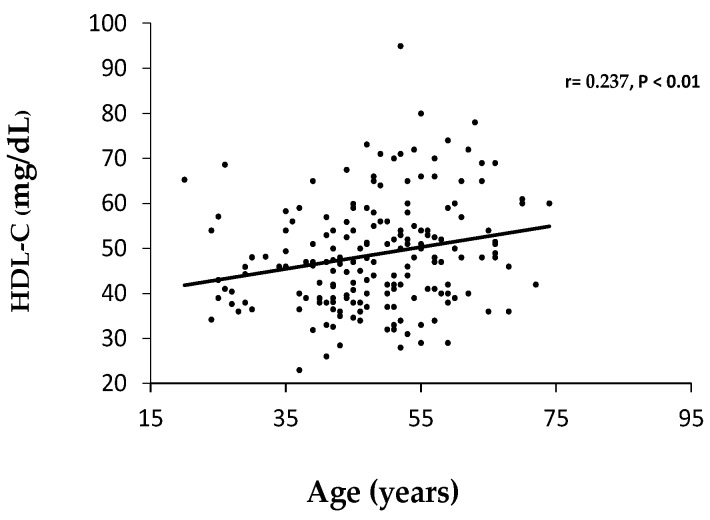
Correlation between age and HDL cholesterol in pre (*n* = 100) and postmenopausal (*n* = 88) women.

In agreement with the results reported in Table 1, significant correlations were found between HDL cholesterol and age for all participants as indicated in Figure 1. These findings suggest that low HDL-C is a more important parameter for MetS classification in the younger women than in those undergoing menopause.

Both pre and post-menopausal women were classified according to HDL cholesterol distribution into: low HDL (<50 mg/dL) and high HDL (≥50 mg/dL). As indicated in Table 2, menopause did not affect any parameter except for age. However HDL status affected: age, LDL-C, HDL-C and TG. Women with higher HDL were older (*p* < 0.05), had lower LDL-C (*p* < 0.01) and lower plasma TG (*p* < 0.001).

**Figure 2:**
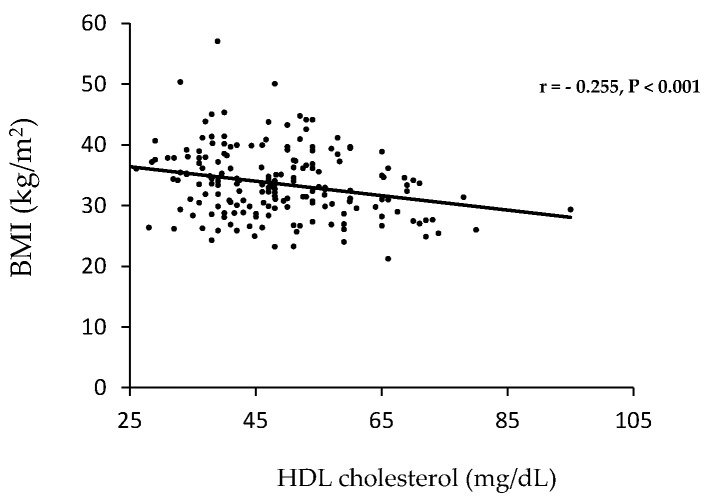
Correlation between HDL cholesterol and body mass index for pre (*n* = 100) and postmenopausal (*n* = 88) women.

**Figure 3:**
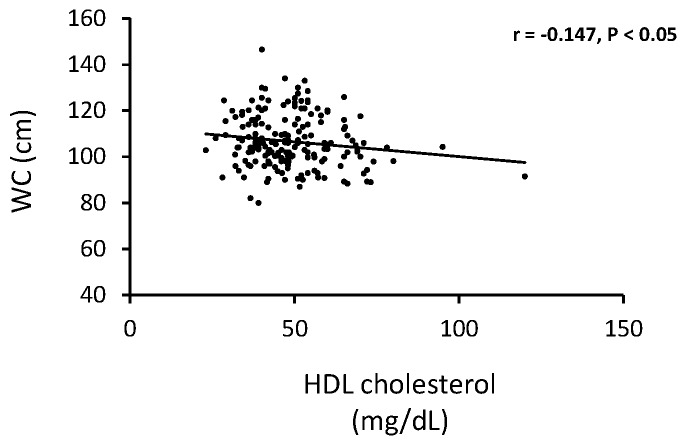
Correlation between HDL cholesterol and waist circumference in 100 pre and 88 post-menopausal women.

Significant negative correlations were found between HDL-C and BMI (Figure 2) and HDL-C and WC (Figure 3) for all subjects suggesting that accumulation of body fat has a detrimental effect on plasma HDL-C.

## 4. Discussion

Menopause is characterized by increases in a number of biomarkers that are associated with higher risk for heart disease and T2DM including visceral adiposity [10,14,33], diastolic and systolic BP [34,35], plasma glucose [8,36], atherogenic lipoproteins [8] and inflammatory markers [8,37]. Some of these biomarkers are part of the criteria for MetS, which explains why the overall prevalence for this condition in the United States goes from 22.6% to over 50% after menopause [38,39]. It is a general understanding that menopause also causes a decrease in the concentration of HDL-C [8,14,36,40], which is another component of MetS [41]; however, in this study we demonstrated that HDL-C is not lower in postmenopausal women when compared with premenopausal women with MetS. In fact, HDL-C concentrations in this study were higher in post- compared to pre-menopausal women. This interesting finding has also been observed by other investigators [25,26,27]. It is possible that the observed increase in HDL-C in postmenopausal women could be due to a protective mechanism to counterbalance the deleterious effects of biomarkers associated with menopause. However, further studies are needed to confirm this theory.

An important observation in these analyses was that HDL-C was negatively correlated to WC, a biomarker of visceral obesity, as well as to BMI. These correlations have been reported by other investigators in heterogeneous populations [42,43,44,45]. This relationship between WC and HDL-C concentrations can be explained by the changes in fat depots which have an important impact in lipoprotein metabolism [46]. An augmented VAT is positively associated with an increased release of free fatty acids (FFA) into the bloodstream, which among other effects, leads to an increased activity of the hepatic lipase [8]. This amplified activity has been associated with low serum concentrations of HDL-C [46,47] because hepatic lipase could convert large, triglyceride rich HDL particles into small HDL particles, which are cleared more rapidly from the circulation [48].

Abdominal obesity is also reported to be associated with other metabolic complications including insulin resistance, glucose intolerance, small and dense LDL, inflammation, altered cytokine profile and endothelial dysfunction [13] which explain why WC is such a strong predictor of cardiovascular disease, hyperlipidemia and T2DM [49,50]. This relationship between HDL-C and WC also helps explain why some authors found HDL-C concentrations to be lower in in post-menopausal women.

One of the physiological changes associated with menopause besides weight gain is a change in body fat distribution [9], which some authors describe as a transition from a gynecoid to an android pattern of fat depots [3]. This means that after menopause VAT increases significantly, independently of age, which suggests that female fat distribution is influenced by sex hormone concentrations [10,33,51]. It is then possible that low HDL-C concentrations observed by some investigators after menopause are not directly caused by menopause but are a consequence of VAT augmentation commonly observed in post-menopausal women. It is also possible that women in which VAT remains unaltered after menopause would have unchanged or even increased HDL-C concentrations, as it is shown in the present study, supporting the hypothesis that menopause does not decrease HDL-C concentration *per se*.

The high concentrations of HDL-C as well as the number of HDL particles have been correlated with numerous healthy benefits not just the well-established role of HDL in RCT [23,52,53]. For example, HDL has been shown to carry important antioxidants in plasma including the carotenoids lutein and zeaxanthin [54], paraoxonase 1, platelet activating factor acetyl-hydrolase, glutathione peroxidase [22]. These antioxidant activities help reduce oxidative stress and thus, LDL oxidation, which is pivotal to the development of atherosclerosis and subsequent cardiovascular diseases [52,55]. In addition, HDL also carries sphingosine-1-phosphate, a molecule that when associated with HDL, could be responsible for the beneficial effects of these lipoprotein on vasorelaxation, cell survival, cell adhesiveness and angiogenesis [56]. Overall, HDL has shown anti-oxidant, anti-inflammatory, anti-thrombotic and anti-apoptotic properties which is why dietary or pharmacological strategies to increase this lipoprotein and its functionality are so important in the prevention of cardiovascular diseases and other metabolic morbidities [22,23,24].

Furthermore, all the women analyzed in this study, whether pre- or post-menopausal with normal values for HDL-C (≥50 mg/dL), also presented lower concentrations of both LDL-C and TG constituting overall a more favorable lipoprotein profile [57]. Thus, VAT and body weight appear to have a greater influence on the dyslipidemias associated with MetS, independent of age and hormonal status. Although these conclusions may have been reached in other analysis, what makes this study unique is the comparisons between pre- and post-menopausal women and the confirmation that HDL-C is not a major risk factor for the older population.

## 5. Limitations

One of the limitations of this study is that the analysis was conducted exclusively with women with MetS, which only describes the phenomena of HDL and menopause in a context of metabolic abnormalities where low HDL is one of the criteria. In addition, the other symptoms of MetS are variable and each individual parameter can affect HDL-C differently. Further studies are needed to compare HDL-C at different ages in other populations, for example, healthy, diabetic or overweight individuals.

Another limitation is that there are techniques available to measure HDL functionality [58] however, the present manuscript does not report HDL functionality, only HDL-C concentrations. Studies have reported that absolute HDL concentrations are poor indicators of HDL functional capacity [59]. This is important because some patients with elevated HDL-C concentrations could remain at risk for coronary events if HDL is not functional [60,61] and some authors have suggested that this could be the case for menopausal women [62].

## 6. Conclusions

From these data, we conclude that women with increased weight and visceral fat are at a higher risk for developing dyslipidemias characterized by high TG and low HDL-C. The lower HDL-C concentrations, which are normally related to increased oxidative stress and inflammation, were more prominent in premenopausal women suggesting that younger women with MetS are at increased risk for heart disease and possibly diabetes than their older counterparts. For women with MetS, WC and BMI are more accurate predictors of HDL-C concentrations, and thus, cardiovascular risk, than menopausal status.

## Figures and Tables

**Table 1:** Anthropometrics, blood pressure plasma lipids and plasma glucose of premenopausal and postmenopausal women with metabolic syndrome ^1^.

Parameter	Premenopausal Women (*n* = 100)	Postmenopausal Women (*n* = 88)	*p* Value
Age (years)	40.7 ± 7.9	57.4 ± 5.8	*p* < 0.001
Number of MetS Parameters	3.4 ± 0.6	3.3 ± 0.6	NS ^2^
Body Weight (kg)	90.0 ± 13.5	88.4 ± 17.5	NS
BMI (kg/m^2^)	34.0 ± 5.3	33.0 ± 6.1	NS
WC ^3^ (cm)	105.6 ± 10.7	107.6 ± 12.0	NS
Systolic BP ^4^ (mm Hg)	128.2 ± 15.6	125.6 ± 15.2	NS
Diastolic BP (mm Hg)	81.3 ± 9.7	81.7 ± 8.5	NS
TC ^5^ (mg/dL)	216.6 ± 34.4	216.1 ± 37.7	NS
LDL-C (mg/dL)	136.9 ± 33.3	134.6 ± 35.2	NS
HDL-C (mg/dL)	46.7 ± 10.7	51.7 ± 14.9	*p* < 0.01
TG ^6^ (mg/dL)	163.9 ± 62.5	167.5 ± 67.0	NS
Glucose (mg/dL)	96.5 ± 12.3	99.4 ± 13.7	NS
Low HDL ^7^ (%)	67%	46.5%	*p* < 0.01

^1^: Values are presented as mean ± SD for the number of participants indicated in parentheses; *p* values were determined by use of non-paired *t*-test; ^2^: Non-significant; ^3^: waist circumference; ^4^: blood pressure; ^5^: total cholesterol; ^6^: triglycerides; ^7^: HDL < 50 mg/dL.

**Table 2:** Distribution of pre and post-menopausal women into Low HDL and High HDL groups ^1^.

	Pre-Menopausal Women		Post-Menopausal Women		*p* Value	
Parameter	Low HDL (*n* = 67)	High HDL (*n* = 33)	Low HDL (*n* = 41)	High HDL (*n* = 47)	Meno-Pause Effect	Low HDL Effect
Age (years)	39.8 ± 7.3	42.6 ± 8.7	57.1 ± 5.7	57.7 ± 5.9	<0.001	0.05
Body Weight (kg)	89.7 ± 12.9	91.0 ± 15.1	89.1 ± 17.4	87.8 ± 17.7	NS ^2^	NS
BMI (kg/m^2^)	34.3 ± 5.4	33.6 ± 6.2	33.7 ± 6.2	32.3 ± 6.0	NS	NS
WC ^3^ (cm)	105 ± 10	107 ± 12	109 ± 12 ^a^	106 ± 12 ^b^	NS	0.01
Systolic BP ^4^ (mm Hg)	130 ± 15	126 ± 16	126 ± 16	126 ± 15	NS	NS
Diastolic BP (mm Hg)	81.4 ± 10.3	81.7 ± 8.5	81.4 ± 9.7	82.9 ± 8.7	NS	NS
TC ^5^ (mg/dL)	216 ± 33	220 ± 32	217 ± 37	218 ± 36	NS	NS
LDL-C (mg/dL)	142 ± 33 ^a^	126 ± 3 ^b^	140 ± 39 ^a^	130 ± 32 ^b^	NS	0.01
HDL-C (mg/dL)	42.7 ± 5.6 ^a^	58.9 ± 7.1 ^b^	40.3 ± 6.2 ^a^	61.7 ±13.0 ^b^	NS	<0.001
TG ^6^ (mg/dL)	167 ± 63 ^a^	171 ± 49 ^a^	202 ± 67 ^a^	138 ± 52 ^b^	NS	<0.001
Glucose (mg/dL)	97± 11	96 ± 14	101 ± 16	99 ± 11	NS	NS

^1^: Values are presented as mean ± SD for the number of participants indicated in parentheses; *p* values were calculated by use of two-way ANOVA. Numbers with different superscripts (a, b) indicate that there is a significant interaction effect; ^2^: Non-significant; ^3^: waist circumference; ^4^: blood pressure; ^5^: total cholesterol; ^6^: triglycerides.

## Abbreviations

The following abbreviations are used in this manuscript:

BMI	body mass index
BP	blood pressure
FBG	fasting blood glucose
FFA	free fatty acids
HDL	high density lipoprotein
HDL-C	HDL cholesterol
LDL-C	LDL cholesterol
MetS	metabolic syndrome
RTC	reverse cholesterol transport
TG	triglycerides
VAT	visceral adipose tissue
WC	waist circumference
